# Renal Thrombotic Microangiopathy in Mice with Combined Deletion of Endocytic Recycling Regulators EHD3 and EHD4

**DOI:** 10.1371/journal.pone.0017838

**Published:** 2011-03-09

**Authors:** Manju George, Mark A. Rainey, Mayumi Naramura, Kirk W. Foster, Melissa S. Holzapfel, Laura L. Willoughby, GuoGuang Ying, Rasna M. Goswami, Channabasavaiah B. Gurumurthy, Vimla Band, Simon C. Satchell, Hamid Band

**Affiliations:** 1 Eppley Institute for Research in Cancer and Allied Diseases, University of Nebraska Medical Center, Omaha, Nebraska, United States of America; 2 Department of Pharmacology, Creighton University, Omaha, Nebraska, United States of America; 3 Department of Pathology and Microbiology, College of Medicine, University of Nebraska Medical Center, Omaha, Nebraska, United States of America; 4 Oncology Central Laboratory, Tianjin Medical University Cancer Institute and Hospital, Tianjin, China; 5 Abbott Laboratories, Abbott Park, Illinois, United States of America; 6 Department of Genetics, Cell Biology and Anatomy, College of Medicine, University of Nebraska Medical Center, Omaha, Nebraska, United States of America; 7 Academic Renal Unit, University of Bristol, Bristol, United Kingdom; 8 Departments of Biochemistry and Molecular Biology, and Pharmacology and Experimental Neuroscience, College of Medicine, University of Nebraska Medical Center, Omaha, Nebraska, United States of America; King's College London, United Kingdom

## Abstract

Eps15 Homology Domain-containing 3 (EHD3), a member of the EHD protein family that regulates endocytic recycling, is the first protein reported to be specifically expressed in the glomerular endothelium in the kidney; therefore we generated *Ehd3* ^–/–^ mice and assessed renal development and pathology. *Ehd3* ^–/–^ animals showed no overt defects, and exhibited no proteinuria or glomerular pathology. However, as the expression of EHD4, a related family member, was elevated in the glomerular endothelium of *Ehd3* ^–/–^ mice and suggested functional compensation, we generated and analyzed *Ehd3* ^–/–^; *Ehd4* ^–/–^ mice. These mice were smaller, possessed smaller and paler kidneys, were proteinuric and died between 3–24 weeks of age. Detailed analyses of *Ehd3* ^–/–^; *Ehd4* ^–/–^ kidneys demonstrated thrombotic microangiopathy (TMA)-like glomerular lesions including thickening and duplication of glomerular basement membrane, endothelial swelling and loss of fenestrations. Other changes included segmental podocyte foot process effacement, mesangial interposition, and abnormal podocytic and mesangial marker expression. The glomerular lesions observed were strikingly similar to those seen in human pre-eclampsia and mouse models of reduced VEGF expression. As altered glomerular endothelial VEGFR2 expression and localization and increased apoptosis was observed in the absence of EHD3 and EHD4, we propose that EHD-mediated endocytic traffic of key surface receptors such as VEGFR2 is essential for physiological control of glomerular function. Furthermore, *Ehd3* ^–/–^; *Ehd4* ^–/–^ mice provide a unique model to elucidate mechanisms of glomerular endothelial injury which is observed in a wide variety of human renal and extra-renal diseases.

## Introduction

Human kidneys filter about 180 liters of blood per day, retaining most of the plasma proteins in blood and allowing passage of water and small molecules into urine. The filtering unit of the kidney, the glomerulus, consists of a network of capillaries covered by specialized visceral epithelial cells, the podocytes, and supported by the mesangial cells in the interstitium. Glomerular capillaries are lined by very specialized glomerular endothelial cells (GEnCs) that possess transendothelial pores or “fenestrations” and an overlying negatively charged glycocalyx [1]. GEnCs and podocytes secrete basement membranes which fuse as glomeruli mature to form the glomerular basement membrane (GBM) [2], [3]. Collectively, GEnCs, the GBM and podocytes together constitute the glomerular filtration barrier [4]–[6]. Injury to or disruption of any of its constituents compromises the integrity of the glomerular filtration barrier resulting in proteinuria. In the recent years, several critical molecular components of podocytes that are indispensable for glomerular filtration have been identified. A number of human diseases with mutated podocyte components have also been identified and several mouse models have been generated that have facilitated our understanding of podocyte biology [7]–[14]. In comparison, our knowledge of molecular components of GEnCs has lagged behind. This is largely due to lack of information on functionally-critical glomerular endothelium-restricted proteins and the unavailability of suitable mouse models that exhibit glomerular disease upon deletion of endothelial-expressed genes.

Members of the EHD protein family (EHD1-4) have emerged as critical regulators of endocytic traffic of membrane as well as cell surface receptors [15]. The reported glomerular endothelium-restricted expression of EHD3 within the kidney [16], [17] suggested the possibility that EHD protein-mediated endocytic recycling might contribute to GEnC-specific functions. This possibility was strengthened by recent studies that have implicated EHD proteins in regulating physiological functions in other cell types, including adipocytes [18], cardiomyocytes [19], myocytes [20], hepatic sinusoidal endothelial cells [21] and neuronal cells [22]. Though EHD3 was the first protein shown to be specifically expressed in the glomerular endothelium, to date, its potential role in this context has not been investigated.

EHD proteins are characterized by an EH domain at the C-terminus, a nucleotide binding P-loop near the N-terminus and a central coiled-coiled region. The EH domain mediates protein–protein interactions by binding to the Asn-Pro-Phe (NPF) tri-peptide motif present in interacting proteins and facilitates membrane binding through association with membrane phospholipids [23], [24]. The P-loop binds and hydrolyzes ATP at a very slow rate *in vitro* [25], while the coiled-coiled region participates in oligomerization [26].

To investigate the *in vivo* functional roles of EHD proteins we have previously employed a gene knockout approach and such studies of *Ehd1* and *Ehd4* have demonstrated their roles in normal murine development and physiology [27]–[28]. Deletion of *Ehd1* resulted in small testis with male infertility (27), while *Ehd4* deletion resulted in small testis with moderate reduction in sperm count (28), indicating a role for EHD1 and EHD4 in male germ cell development/differentiation. Deletion of *Ehd1* also leads to embryonic lethality and ocular defects whose severity varies with the genetic background of the mouse strains used (Rainey MA *et al*., manuscript in preparation). Here, we extended this gene knockout approach to *Ehd3*; however, *Ehd3* ^–/–^ mice showed no discernable pathology. Upregulation of the expression of EHD4, a related family member, in *Ehd3* ^–/–^ GEnCs led us to hypothesize that EHD4 functionally compensates for loss of EHD3. We generated *Ehd3^–/–^; Ehd4^–/–^* mice to test this hypothesis and observed severe glomerular disease in these mice, demonstrating a critical role for EHD3 and EHD4 in glomerular health. This is the first report of a knockout mouse model where deletion of genes with GEnC-enriched expression results in phenotypes similar to human diseases with glomerular endothelial injury as the initiating event.

## Results

### Generation and characterization of Ehd3^–/–^ mice

A recombineering strategy similar to that successfully used to generate *Ehd1* and *Ehd4* null mice in our earlier studies [27], [28], was employed to target the 5′untranslated region and the first exon of *Ehd3* such that targeted animals would not express EHD3 (Text S1 and Figure S1A). Genotyping by PCR (Figure S1B) and Western blotting of organ lysates (Figure S1C) from wild-type (*Ehd3* ^+/+^), heterozygote (E*hd3* ^+/–^) and homozygous deleted (*Ehd3* ^–/–^) mice confirmed successful targeting. *Ehd3* ^–/–^ mice were born at expected Mendelian ratios, were healthy and fertile with body weights comparable to their wild-type littermates (Figure S1D & S1E).

### Developmental regulation of EHD3 expression in renal glomerular endothelium

**Figure 1:**
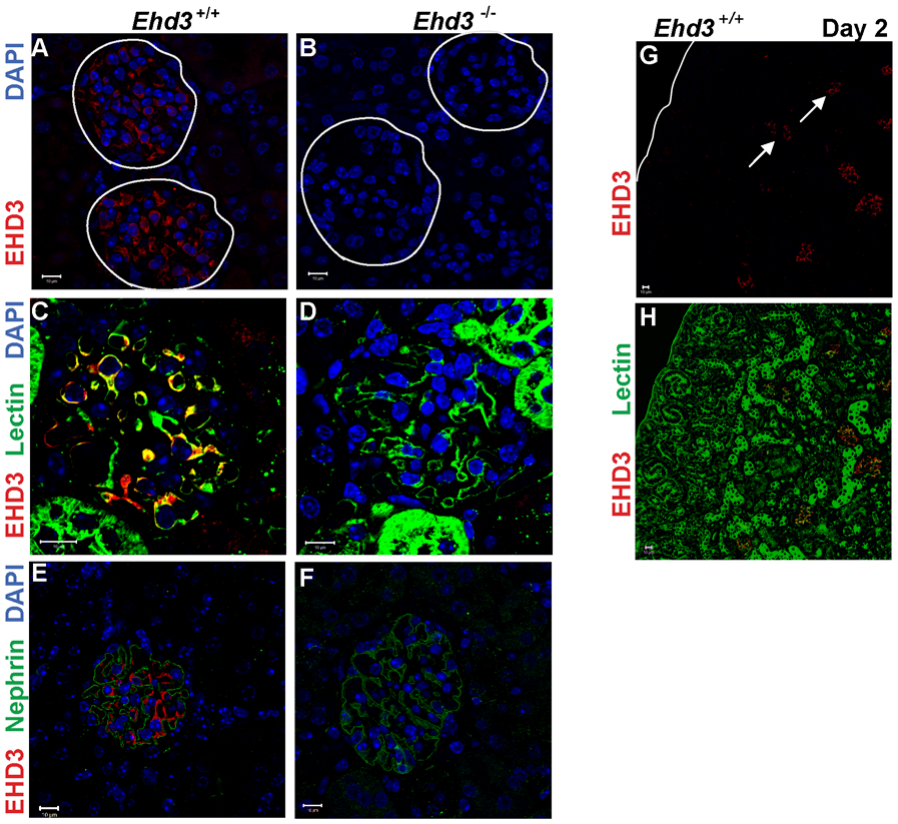
EHD3 expression in the glomerular endothelium. (A–B) Three µm thick kidney sections from three month old male *Ehd3^+/+^* and *Ehd3^–/–^* mice were stained using antibodies to EHD3 and confocal images acquired as described. EHD3 staining (red) and DAPI staining of nuclei (blue) are shown. White circles demarcate glomeruli. (C–D) Triple staining of kidney sections with labeled tomato lectin (green) and EHD3 (red) and DAPI (blue) shows colocalization between EHD3 and tomato lectin staining (yellow in panel C). (E–F) Triple staining with the podocyte marker, nephrin (green), EHD3 (red) and DAPI (blue) shows nephrin staining around endothelial EHD3 staining in panel E. EHD3 staining is absent in *Ehd3^–/–^* kidney sections as expected in panels B, D and F. (G–H) Kidney sections from 2 day old *Ehd3^+/+^* mice were double stained with EHD3 (red) and lectin (green), yellow in panel H shows colocalization. The white line demarcates the kidney capsule in panel G and white arrows point to EHD3 expression in capillary loop stage glomeruli. S-shaped and comma shaped glomeruli that lack EHD3 staining are clearly visible in the nephrogenic zone in panel H. Scale bar = 10 µm.

As lack of a renal phenotype in *Ehd3* ^–/–^ mice was unexpected, we first confirmed that EHD3 is indeed expressed in the wild-type kidney using *Ehd3* ^–/–^ kidney as a negative control. Consistent with a previous report [16], our analysis of adult *Ehd3* ^+/+^ mouse kidney sections confirmed predominant EHD3 expression (Figure 1A) in GEnCs as confirmed by co-staining of these cells with endothelial markers tomato lectin (from *Lycopersicon esculentum)* (Figure 1C) and CD31 (data not shown)*;* tomato lectin recognizes N-acetyl glucosamine (GlcNAc) and poly-N-acetyllactosamine on the glomerular endothelium [29]. EHD3 staining was not seen in podocytes which stained strongly with the podocyte protein nephrin, (Figure 1E); nephrin stained podocytes form a discrete rim surrounding EHD3-positive cells, further confirming EHD3 expression in GEnCs. The absence of staining in *Ehd3* ^–/–^ glomeruli validated the specificity of GEnC-specific EHD3 expression (Figure 1B, D and F).

We next assessed if EHD3 expression in GEnCs is developmentally regulated. Nephrogenesis in mice begins by embryonic day 11 [3] and glomerular development proceeds through a series of nephric structures morphologically seen as vesicle, comma- and S-shaped, developing capillary loop, and maturing glomerulus stages. Newborn mice show a sub-cortical “nephrogenic zone” with easily discernable vesicular, comma and S-shaped nephric structures and an expanding cortex with capillary loop and maturing glomerular stages [30]. Analyses of *Ehd3* ^+/+^ kidneys at postnatal day 2 showed EHD3 expression in the capillary loop stage and mature glomeruli (Figure 1G and H) but not in the earlier nephric stages, indicating that its expression is indeed developmentally regulated.

In view of the endothelium-restricted and developmentally-regulated expression of EHD3 in the kidney, we undertook detailed analyses of *Ehd3* ^–/–^ kidneys to characterize potentially subtle glomerular pathology. However, no detectable glomerular abnormalities were observed in H&E and Periodic Acid Schiff (PAS) stained *Ehd3* ^–/–^ kidney sections (Figure S2A–D). Immunofluorescence microscopy showed similar patterns of staining of endothelial, mesangial and podocyte markers in both *Ehd3* ^+/+^ and *Ehd3* ^–/–^ glomeruli (Figure S2F–M) and transmission electron microscopic (TEM) analysis of *Ehd3* ^–/–^ kidneys from 8-month old mice showed glomerular endothelial cells with normal fenestrations and intact podocyte foot processes (Figure S2N–O). Finally, no proteinuria was detected in urine samples from *Ehd3* ^–/–^ mice on SDS-PAGE (Figure S2E) indicating that the functional integrity of the GBM was unaffected in *Ehd3* ^–/–^ kidneys.

### Compensatory increase in EHD4 expression in Ehd3^–/–^ glomerular endothelium

**Figure 2:**
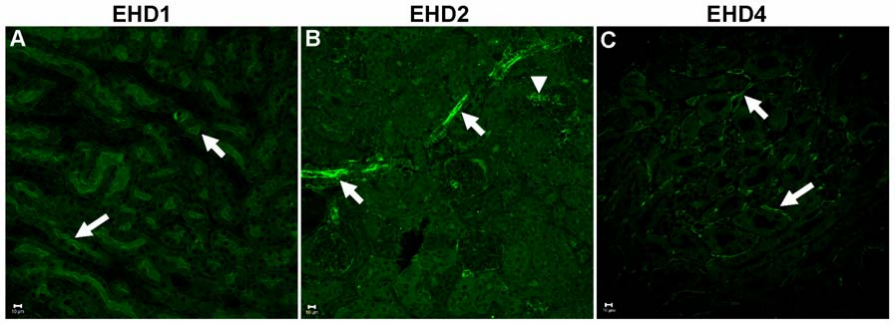
Spatially distinct expression of EHD proteins in the kidney. (A–C) Kidney sections from three month old male *Ehd3^+/+^* and *Ehd3^–/–^* mice were processed and stained using antibodies to indicated EHD proteins and confocal images acquired as described in Materials and Methods. EHD staining is shown in green. Scale bar = 10 µm. White arrows point to EHD1 expression in brush border epithelium (panel A), EHD2 expression in the interlobular arteries (panel B), and EHD4 expression in the peritubular capillaries (panel C); the white arrow head points to EHD2 expression in afferent arterioles (panel B).

Complete absence of glomerular pathology in *Ehd3* ^–/–^ kidneys despite GEnC-enriched EHD3 expression could reflect a lack of requirement of EHD3 in glomerular development and function, or compensatory upregulation of other EHD proteins in the *Ehd3* ^–/–^ GEnCs. As no information is available on the relative expression of other EHD proteins in the kidney, we undertook immunostaining analyses to assess if other EHD family members are also expressed in the glomerular endothelium. Immunostaining for EHD1, EHD2 and EHD4 showed relatively low signals in the glomerular endothelium but markedly more prominent staining in spatially distinct regions of the kidney: EHD1 was expressed in the brush-border epithelium of proximal tubules (white arrows in Figure 2A); EHD2 in the endothelium and smooth muscle cells of interlobular arteries (white arrows, Figure 2B) and afferent arterioles (white arrow heads in Figure 2B); and EHD4 in the peritubular capillary endothelium (white arrows in panel Figure 2C). *Ehd1* ^–/–^ and *Ehd4* ^–/–^ kidney sections were used as controls for EHD1 and EHD4 staining, respectively (data not shown); staining without a primary antibody was used as a control for EHD2 staining. Thus, as EHD3 appears to be the predominant GEnC-enriched EHD protein, it is unlikely that lack of glomerular defects in *Ehd3* ^–/–^ mice is due to basal co-expression of other EHD family members.

**Figure 3:**
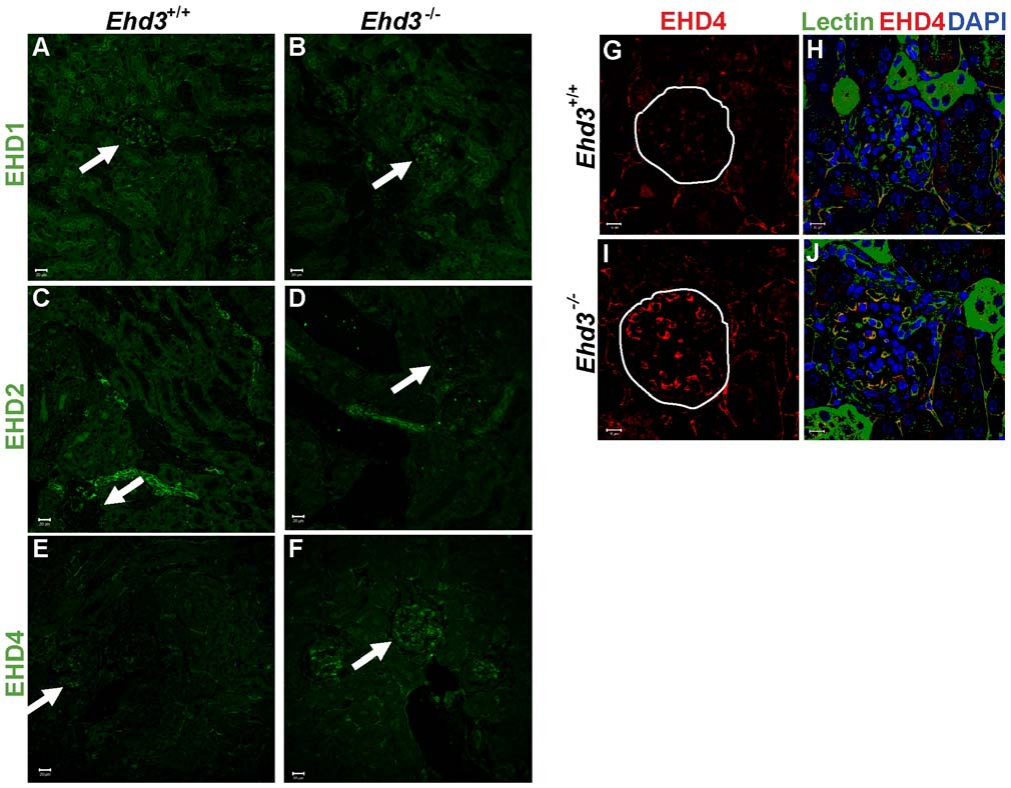
Compensatory increase in EHD4 in the *Ehd3^–/–^* glomerular endothelium. (A–B) Confocal images of formalin-fixed, paraffin-embedded 3 µm thick kidney sections from 5 month old male *Ehd3* ^+/*+*^ and *Ehd3^–/–^* mice were immunostained with antibodies to EHD1, (C–D) EHD2 and (E–F) EHD4. EHD protein staining is shown in green. One glomerulus in each panel is denoted by a white arrow. Scale bar = 20 µm. (G–J) Sections triple stained for EHD4 (red, panel G–J), labeled tomato lectin (green) and DAPI (blue) are shown. Yellow in panel J shows colocalization between lectin and EHD4. White circles demarcate glomeruli. Scale bar = 10 µm.

As previous studies have shown compensatory changes in EHD protein expression upon knockout of individual family members [27]–[28], [31] we examined *Ehd3* ^–/–^ kidneys for upregulation of other EHD protein expression. Immunofluorescence analyses showed lack of appreciable differences in EHD1 or EHD2 expression between *Ehd3* ^+/+^ and *Ehd3* ^–/–^ kidney sections (Figure 3A–B and 3C–D). Notably, while EHD4 expression in the peritubular capillaries was comparable in *Ehd3* ^+/+^ and *Ehd3* ^–/–^ kidney sections, a marked increase in EHD4 expression was observed in *Ehd3* ^–/–^ glomeruli (Figure 3E–F). Co-staining with labeled tomato lectin showed that EHD4 was specifically upregulated in the *Ehd3* ^–/–^ GEnCs (Figure 3G–J). The selective upregulation of EHD4 in *Ehd3* ^–/–^ GEnCs suggested that EHD4 might functionally compensate for *Ehd3* deletion and account for lack of glomerular pathology.

### Combined deletion of Ehd3 and Ehd4 in mice leads to renal pathology with proteinuria

**Figure 4:**
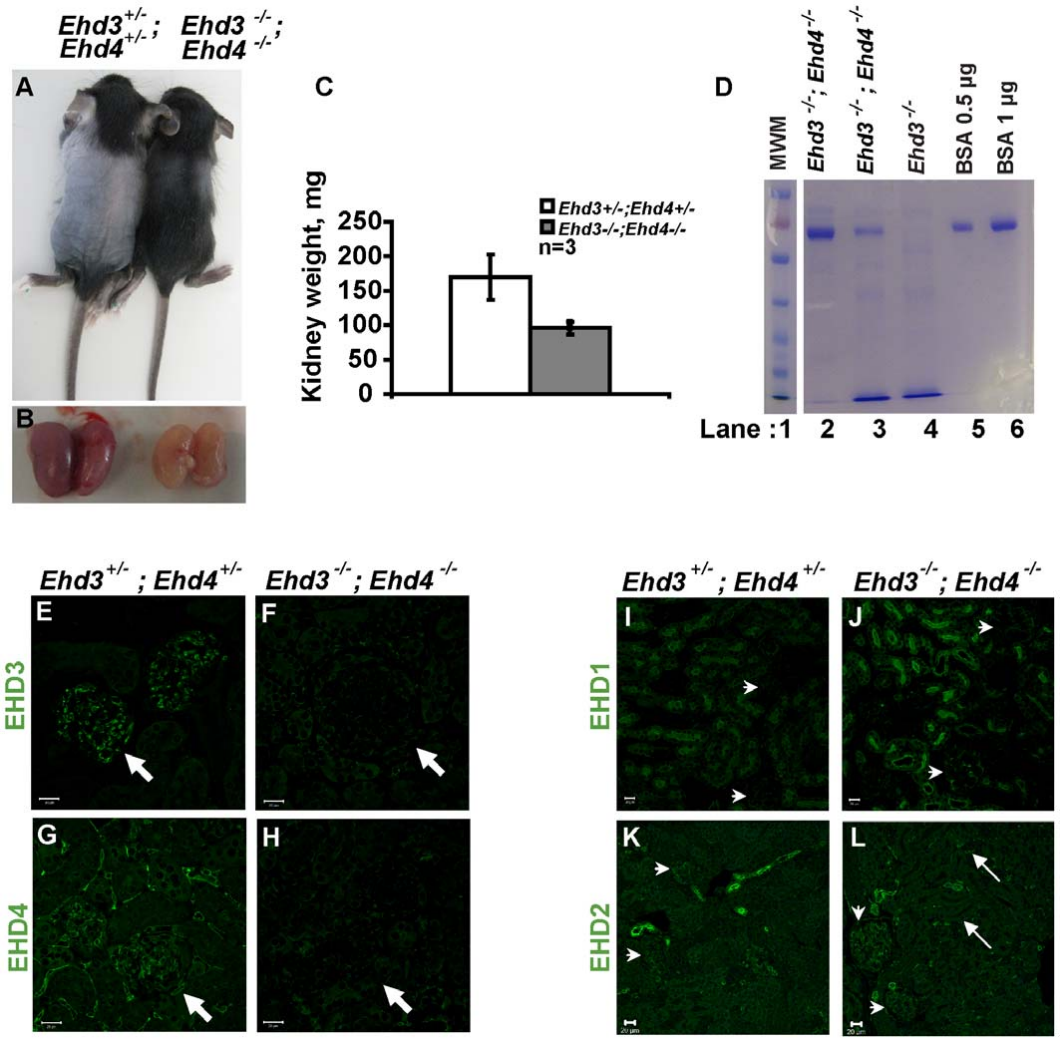
Proteinuria in *Ehd3* ^–/–^ *; Ehd4* ^–/–^ mice. (A) Seventeen day-old *Ehd3* ^–/–^ *; Ehd4* ^–/–^ and littermate *Ehd3* ^+/–^ *; Ehd4* ^+/–^ mice were euthanized and photographed. (B) Kidneys dissected from mice shown in (A) were photographed, note the smaller and paler *Ehd3* ^–/–^ *; Ehd4* ^–/–^ kidneys. (C) Kidneys weights from 3-week old *Ehd3* ^–/–^ *; Ehd4* ^–/–^ mice and littermate controls were plotted, error bars indicate standard deviation. (D) Urine samples from 17 day old mice of indicated genotypes (lane 2 & 3) and a 3 month old *Ehd3* ^–/–^ mouse (lane 4) were run on a 7.5% SDS-PAGE and stained with Coomassie Blue. Gels were scanned following de-staining. Bovine serum albumin (BSA) was used as a positive control (lanes 5 and 6). MWM, molecular weight marker (lane 1). Lanes 1–6 were run on the same gel but were noncontiguous. (E–H) 3 µm kidney sections from mice shown in (A) were stained for EHD3 and EHD4 (green) and confocal images acquired as described. White arrows point to expression of EHD3 and EHD4 in *Ehd3* ^+/–^ *; Ehd4* ^+/–^ kidney sections (panel E and G) and absence of expression in *Ehd3* ^–/–^ *; Ehd4* ^–/–^ kidney sections (panel F and H). (I–L) Kidney sections from mice shown in (A) were stained for EHD1 (green, panels I–J) and EHD2 (green, panels K–L) and confocal images acquired as described. White arrows point to expression of EHD1 and EHD2 in kidney sections, while white arrow heads point to glomeruli. Scale bar = 20 µm.

To test the hypothesis that a compensatory increase in EHD4 expression prevents the appearance of glomerular pathology in *Ehd3* ^–/–^mice, we intercrossed *Ehd3* ^–/–^ mice with the previously described *Ehd4* ^–/–^ mice [28], [31] to generate *Ehd3* ^–/–^ *; Ehd4* ^–/–^ double-null mice. *Ehd3* ^–/–^ *; Ehd4* ^–/–^ mice were smaller (Figure 4A), displayed markedly pale (Figure 4B) and smaller kidneys (Figure 4C), developed severe proteinuria (Figure 4D) and died between 3–24 weeks of age. As expected, both EHD3 and EHD4 staining was absent in *Ehd3* ^–/–^ *; Ehd4* ^–/–^ kidneys (Figure 4E–H). Interestingly, EHD2 expression was upregulated in the glomerular and peritubular capillary endothelium of *Ehd3* ^–/–^ *; Ehd4* ^–/–^ kidneys (Figure 4K–L). *Ehd3* ^–/–^ *; Ehd4* ^–/–^ mice did not show an upregulation of EHD1 in the glomerular or peritubular capillary endothelium (Figure 4I–J), but showed increased EHD1 expression at the proximal tubule brush border epithelium. Overall, these results indicate that concurrent deletion of *Ehd3* and *Ehd4* leads to renal pathology.

### Ehd3^–/–^; Ehd4^–/–^ mice develop thrombotic microangiopathy

**Figure 5:**
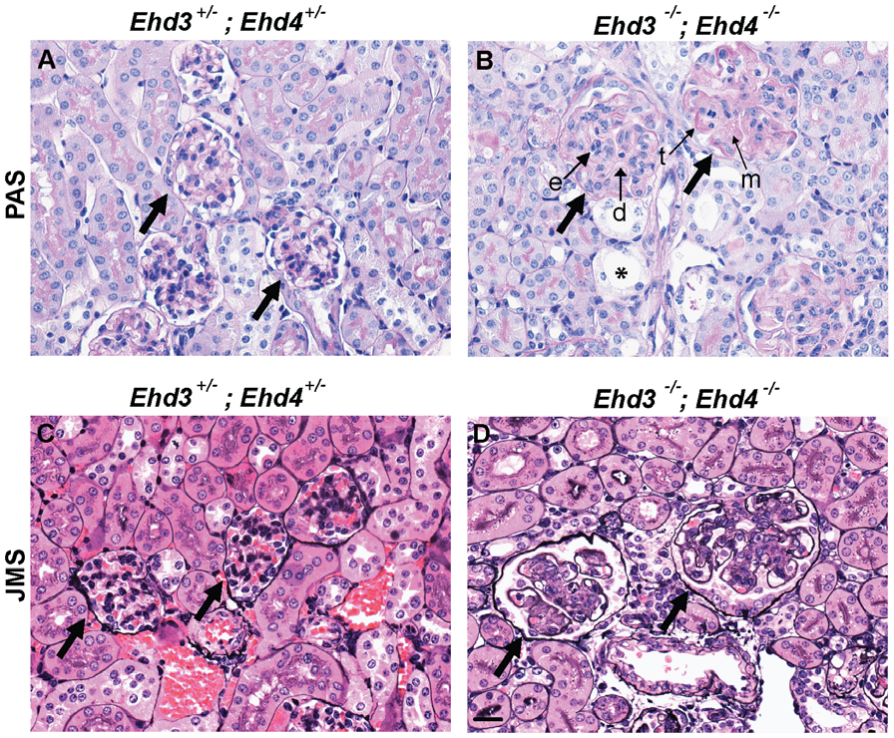
*Ehd3^–/–^; Ehd4^–/–^* mice develop thrombotic microangiopathy. (A–B) Kidney sections from 22 day old *Ehd3^–/–^; Ehd4^–/–^* mice and littermate controls were stained with PAS to visualize tubular and glomerular basement membranes. Healthy capillary loops are seen in the *Ehd3* ^+/–^; *Ehd4* ^+/–^ glomeruli (panel A) while *Ehd3^–/–^; Ehd4^–/–^* kidney sections showed endothelial swelling (e), enlarged glomeruli with segmental thickening (t), duplication of basement membranes (d) and mesangiolysis (m). Some tubular dilation is also seen (asterisks). (C–D) Jones methenamine silver (JMS) staining of kidney sections from *Ehd3^–/–^; Ehd4^–/–^* mice (panel D) and littermate controls (panel C) show duplicated and thickened glomerular basement membrane, expanded mesangium and relatively avascular glomeruli in *Ehd3^–/–^; Ehd4^–/–^* kidney sections. Thick black arrows point to glomeruli.

H&E and PAS staining of kidney sections from *Ehd3^–/–^; Ehd4^–/–^* mice showed lesions characteristic of thrombotic microangiopathy (TMA). TMA is a lesion observed in a number of human diseases including pre-eclampsia, hemolytic uremic syndrome and malignant hypertension and is defined by the primary locus of injury in the endothelium [32]. Although the majority of human cases of TMA exhibit variable numbers of thrombi, a percentage of cases do not. For example, pre-eclampsia, which has the characteristic lesions of TMA, rarely exhibits thrombi. A diagnosis of TMA is based on a set of characteristic morphologic findings irrespective of the presence of glomerular or arteriolar thrombi. These lesions include endothelial cell swelling, expansion of the subendothelial zone, duplication of the GBM, mesangial cell interposition and evidence of red cell destruction. PAS stained *Ehd3^–/–^; Ehd4^–/–^* mouse kidney sections showed characteristic glomerulomegaly, thickening and duplication of the GBM, expanded and lytic-appearing mesangium, variable degrees of mesangial interposition and abnormal capillary loops (Figure 5A–B). Other changes included tubular dilation and protein reabsorption droplets in proximal tubules. The glomerular changes were more easily visible with the Jones Methenamine silver stain (Figure 5C–D). Notably, we did not observe any thrombi in the *Ehd3^–/–^; Ehd4^–/–^* mouse kidney sections analyzed despite other hallmarks of TMA.

**Figure 6:**
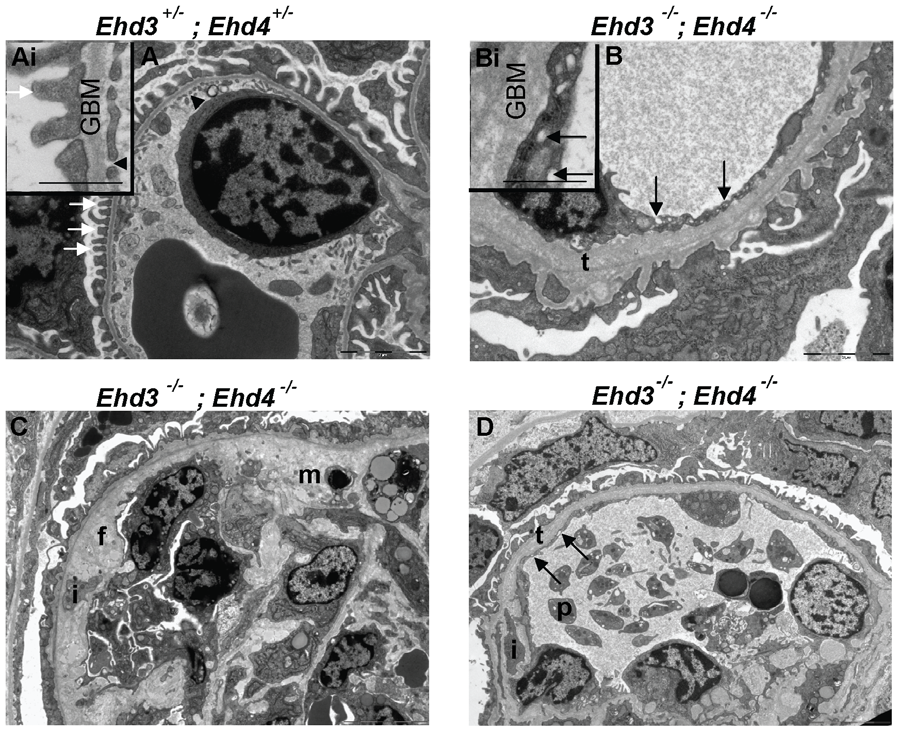
Ultrastructural changes in *Ehd3^–/–^; Ehd4^–/–^* glomeruli. (A) Endothelial cells with fenestrations (black arrow heads) and podocytes with intact foot processes (white arrows) are evident in the *Ehd3* ^+/–^; *Ehd4* ^+/–^ section. (Ai) Inset shows a representative higher magnification image, inset scale bar = 500 nm. (B) Abnormally thickened and lamellated basement membranes (t), swollen endothelium without fenestrations and effacement of podocytes foot processes were seen in the *Ehd3^–/–^; Ehd4^–/–^* kidney section. Multiple small “holes” presumed to be abnormal endosomes were seen in the endothelium (black arrows); these structures are clear in the higher magnification image in B. Inset scale bar = 500 nm. Scale bars = 2 µm. (C–D) Images from *Ehd3^–/–^; Ehd4^–/–^* kidneys also show additional changes including mesangiolysis (m), mesangial interposition (i), flocculent subendothelial material (f) and platelet (p) accumulation in a capillary loop. GBM, glomerular basement membrane. Scale bars = 5 µm.

In order to assess the ultrastructural alterations in glomerular endothelial cells of *Ehd3^–/–^; Ehd4^–/–^* mice, we performed TEM of kidney sections. Endothelial cells with a normal pattern of fenestrations (black arrow heads, Figure 6A and Ai), podocytes with intact foot processes (white arrows, Figure 6A and Ai) and normal GBM were identified in *Ehd3^+/–^; Ehd4^+/–^* kidneys (Figure 6A and Ai) and in kidney sections from *Ehd3^–/–^; Ehd4^+/–^ and Ehd3^+/–^; Ehd4^–/–^* mice (data not shown), indicating that the presence of at least one copy of *Ehd3* or *Ehd4* gene was sufficient to assure ultrastructurally normal glomerular capillaries.

In contrast, TEM of *Ehd3^–/–^; Ehd4^–/–^* kidney sections (Figure 6B–D) revealed swollen glomerular endothelial cells without fenestrations, podocytes with variable segmental foot process effacement and thickening and lamellation of GBM (t, Figure 6B, Bi and D) with widening of subendothelial zones containing “flocculent material” (f, Figure 6C), lucencies within mesangial areas suggesting lysis (m, Figure 6C) and variable mesangial interposition (i, Figure 6C). Many glomerular endothelial cells contained numerous small structures that looked like abnormal endosomes or vacuoles (Figure 6B, Bi and Figure 6D, black arrows). These structures were never observed in kidneys from littermate controls. Other changes observed included electron dense deposits in mesangial areas (data not shown), vacuolation within the mesangium and platelet aggregation (p, Figure 6D) within capillary lumens. It is interesting to note that marked pathology was seen not only in endothelial cells but also in podocytes and mesangial cells within the glomeruli. Characteristic lesions of TMA were seen in all *Ehd3^–/–^; Ehd4^–/–^* kidneys analyzed (at ages of day 17 (d17), d22, d23, d39, d45 and d180), while the only 6 month old animal showed cellular infiltration and collagen deposition as additional renal changes (data not shown). The observed lesions were strikingly similar to that seen in human TMAs like pre-eclampsia and in mouse models with reduction of podocyte-expressed VEGF [32]–[34].

### Staining and distribution of endothelial, podocytic and mesangial markers were drastically altered in Ehd3^–/–^; Ehd4^–/–^ glomeruli

**Figure 7:**
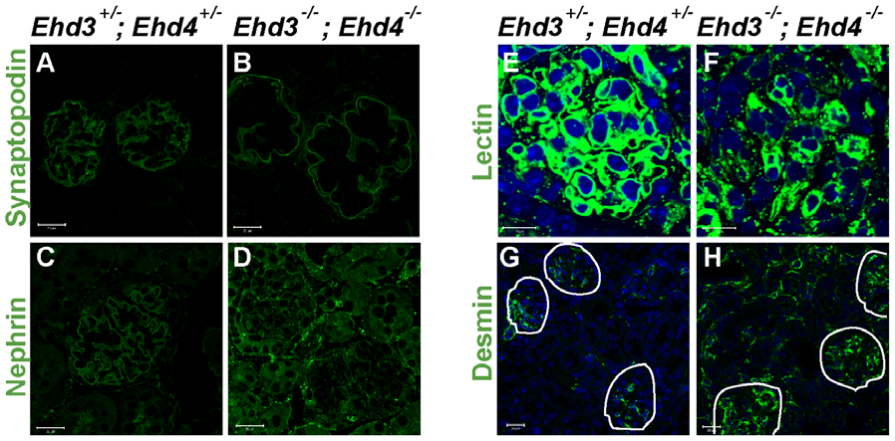
Alteration in endothelial, podocytic and mesangial cells in *Ehd3^–/–^; Ehd4^–/–^* glomeruli. (A–D, G–H) Kidney sections from 39 day old *Ehd3* ^+/–^; *Ehd4* ^+/–^ and *Ehd3^–/–^; Ehd4^–/–^* mice were immunostained with antibodies to synaptopodin (A–B), nephrin (C–D) and desmin (G–H) as described in Materials and Methods. (E–F) Endothelial cells were visualized by labeled tomato lectin staining (green) and DAPI (blue) was used to counterstain nuclei. Markedly altered staining patterns were observed in the *Ehd3^–/–^; Ehd4^–/–^* kidney sections in each case. Scale bar = 20 µm in panels A–F and 10 µm in panels G–H. White lines demarcate glomeruli in panels G–H.

To further characterize the lesions observed by light microscopy and TEM, we performed immunostaining for glomerular cell type markers. The staining patterns of podocyte markers nephrin and synaptopodin were severely altered in *Ehd3^–/–^; Ehd4^–/–^* glomeruli with loss of the lobulated staining pattern seen in control *Ehd3^+/–^; Ehd4^+/–^* glomeruli (Figure 7A–D). Tomato lectin staining of GEnCs and desmin staining of mesangial cells showed markedly altered expression patterns; *Ehd3^–/–^; Ehd4^–/–^* glomeruli had fewer lectin-positive endothelial cells (Figure 7E–F) and desmin expression was greatly upregulated in the glomeruli and interstitium when compared to littermate controls (Figure 7G–H). These studies indicate that concurrent deletion of *Ehd3* and *Ehd4* adversely affects all glomerular cell types.

### Altered localization of VEGFR2 and increased apoptosis in Ehd3^–/–^; Ehd4^–/–^ glomeruli

**Figure 8:**
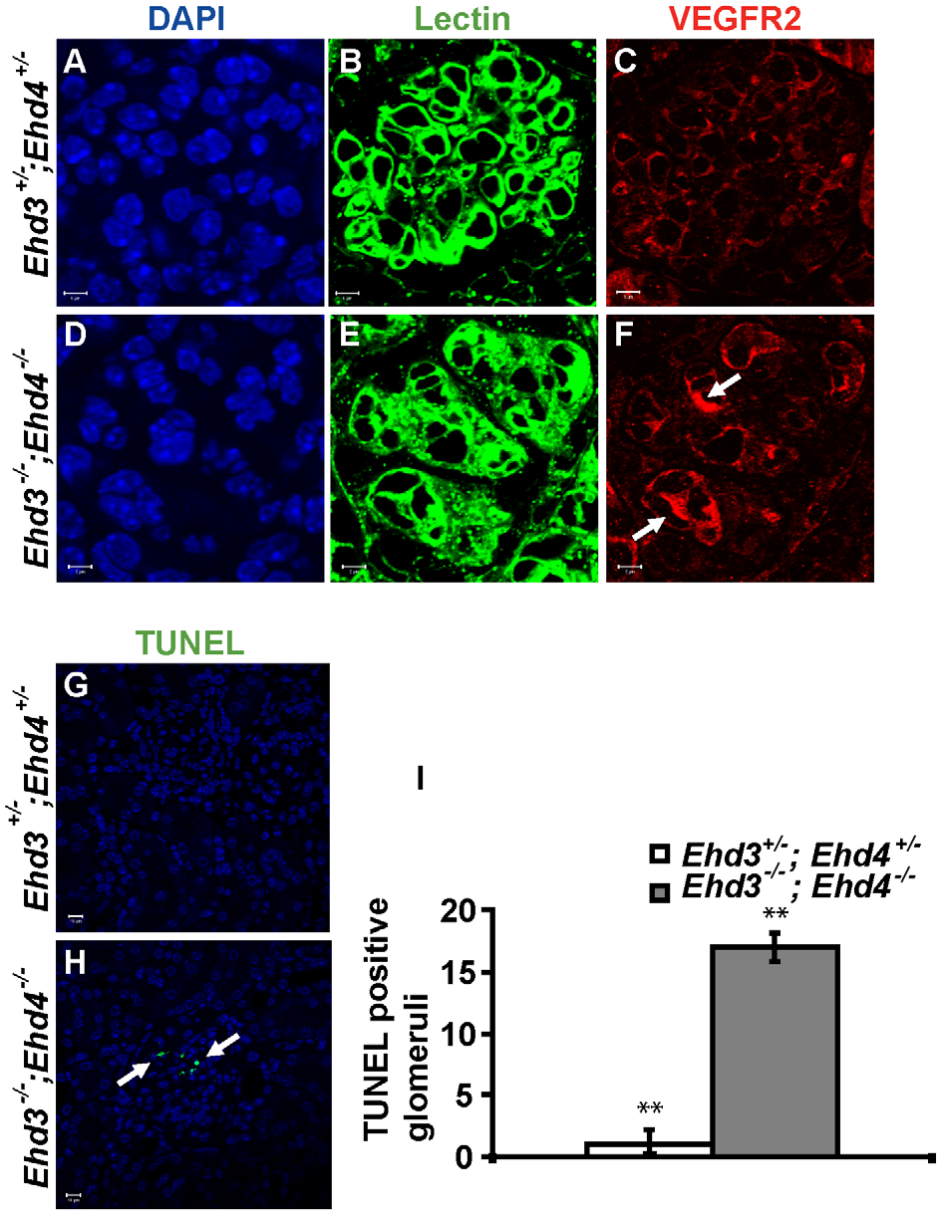
Altered VEGFR2 localization and increased apoptosis in *Ehd3^–/–^*; *Ehd4^–/–^* glomeruli. (A–F) Kidney sections from *Ehd3^+/–^*; *Ehd4^+/–^* and *Ehd3^–/–^*; *Ehd4^–/–^* mice were immunostained with antibodies to VEGFR2 (red). Labeled lectin (green) was used to mark endothelial cells (B and E), while DAPI (blue) stained nuclei (A and D). Uniform low level of VEGFR2 staining in seen in lectin positive glomerular endothelial cells in the *Ehd3^+/–^*; *Ehd4^+/–^* glomeruli (panel C), while diffuse and abnormal lectin staining was seen in the *Ehd3^–/–^*; *Ehd4^–/–^* glomeruli (panel F), few lectin positive cells showed intense VEGFR2 staining (white arrows, panel F). (G–I) Kidney sections from *Ehd3^+/–^*; *Ehd4^+/–^* and *Ehd3^–/–^*; *Ehd4^–/–^* mice were subjected to TUNEL assay as described in Materials and Methods. Confocal images of TUNEL staining (green) are shown (panels G–H), white arrow points to apoptotic nuclei (panel H). About 45 glomeruli each were counted in 12 kidney sections to arrive at the graph (panel I). Error bars indicate standard deviation (**indicates P<0.05 using two-tailed analysis).

Given the similarity of glomerular lesions in *Ehd3^–/–^; Ehd4^–/–^* mice with those seen in mice with 50% reduced podocytic VEGF expression [33], [34] and in pre-eclampsia in humans [32], we analyzed if VEGF, VEGFR1 or VEGFR2 expression was altered in *Ehd3^–/–^; Ehd4^–/–^* glomeruli. While the expression pattern of VEGF and VEGFR1 in *Ehd3^–/–^; Ehd4^–/–^* glomeruli were comparable to those in *Ehd3^+/–^; Ehd4^+/–^* littermate control glomeruli (data not shown), they displayed a dramatically altered expression of VEGFR2 on endothelial cells, as seen upon co-staining for VEGFR2 and tomato lectin (Figure 8A–F). Tomato lectin staining was very diffuse and fewer GEnCs stained positive for VEGFR2 in *Ehd3^–/–^; Ehd4^–/–^* glomeruli; in contrast, nearly all GEnCs showed VEGFR2 staining in control *Ehd3^+/–^; Ehd4^+/–^* glomeruli. Notably, some *Ehd3^–/–^; Ehd4^–/–^* GEnCs showed dramatically more intense intracellular VEGFR2 staining compared to control glomeruli (white arrows, Figure 8F), suggesting potential defects in VEGFR2 trafficking.

In view of the altered expression and distribution of VEGFR2 in *Ehd3^–/–^; Ehd4^–/–^* glomeruli and the known role of VEGF signaling in regulating glomerular physiology [34]–[36], we assessed if *Ehd3^–/–^; Ehd4^–/–^* glomeruli showed increased apoptosis. Indeed TUNEL detected a significantly large increase in apoptotic cells in *Ehd3^–/–^; Ehd4^–/–^* glomeruli compared to *Ehd3^+/–^; Ehd4^+/–^* glomeruli (Figure 8G–I).

## Discussion

Studies reported here describe the generation and characterization of a mouse model in which concurrent deletion of two endocytic recycling regulatory proteins, EHD3 and EHD4, results in renal thrombotic microangiopathy (TMA), a pathological lesion commonly seen in diverse clinical conditions. TMA is also seen in pre-eclampsia, a condition that affects about 5% of all pregnancies and is thus the most common glomerular disease in the world [32]. Irrespective of the clinical condition, the initiating event in TMA is thought to be an insult or injury to the endothelium. Though research in recent years has remarkably increased our understanding of the cell types in the glomeruli, much is still unknown about the glomerular endothelial cell. Whole-body deletion of endothelial-expressed genes in mice often results in embryonic lethality precluding analyses of endothelial cell function in the maturing glomerulus. A limited set of genes have recently been shown to be specifically expressed in the glomerular endothelium [16], [17], however, knockout mouse models of these genes have not been described. The lack of suitable animal models describing glomerular endothelial disease upon genetic deletion of genes specifically expressed in the glomerular endothelium has hampered research into this important cell type in the renal glomerulus. Thus, our studies reporting a gene knockout mouse model that recapitulates features of human TMA represents a major step forward that should facilitate mechanistic and pharmacological explorations of glomerular endothelial injury.

Since we were unable to find unaltered endothelium in the double null mice of various ages analyzed, our results suggest that processes regulated by EHD proteins are critical to glomerular endothelial health. In view of the extensive changes in the endothelial compartment, together with endothelial-specific expression of EHD3 (and increased endothelial EHD4 in *Ehd3^–/–^* glomeruli), it is reasonable to suggest that the changes seen in podocytes and mesangial cells are likely to be secondary to endothelial defects caused by deletion of *Ehd3* and *Ehd4*. This is not unexpected as cell types within the glomerulus communicate via soluble mediators; elegant studies have shown that injury to any one cell type could compromise the functional integrity of other cell types and the glomerulus as a whole [35], [37]–[39]. For example, soluble factors such as platelet derived growth factor (PDGF) BB elaborated by healthy endothelial cells have been shown to be critical to mesangial health [37] and this might explain the mesangial changes seen in *Ehd3^–/–^; Ehd4^–/–^* mice. It is also possible that the observed alterations in VEGFR2 expression in glomerular endothelial cells (Figure 8) might affect their health and secondarily contribute to the podocyte pathology seen. Future studies to delete *Ehd3* and *Ehd4* in a cell type-specific manner (endothelium or podocytes) using specific Cre-recombinase expressing transgenic mouse lines will help unequivocally distinguish the direct versus indirect effects of endothelial EHD3 and EHD4 loss on the podocyte. Towards this end, we have generated double *Ehd3 ^fl/fl^*; *Ehd4 ^fl/fl^* mice that should allow such experiments to be carried out in the future.

The TMA observed in *Ehd3^–/–^; Ehd4^–/–^* glomeruli bear a striking resemblance to that seen in mouse models with reduced podocyte VEGF expression and a recent mouse model with inducible whole-body deletion of VEGFR2 [40]. This is especially significant since our genetic manipulations were not aimed at altering the levels of VEGF or its receptors, VEGFR1 and VEGFR2 expressed in the glomerular endothelium. The marked alterations in VEGFR2 expression in glomerular endothelial cells and the increased apoptosis seen in *Ehd3^–/–^; Ehd4^–/–^*glomeruli, however point to a possible role for EHD proteins in the endocytic traffic of VEGFR2 and subsequent regulation of VEGF mediated signaling in the glomeruli. It has been demonstrated that at steady state VEGFR2 localizes to the cell surface and intracellular vesicles presumed to be sorting endosomes [41]. In response to VEGF addition, recycling of intracellular VEGFR2 to the cell surface increases to allow higher VEGF binding and signaling [41], [42]. Since EHD3 [26] and EHD4 [43] are both known to regulate trafficking out of sorting endosomes, and in view of the appearance of abnormal vesicular structures in endothelial cells as seen by TEM, it is possible that altered recycling of VEGFR2 out of sorting endosomes in *Ehd3^–/–^; Ehd4^–/–^* glomerular endothelial cells might impair VEGF signaling resulting in the phenotypes we observe. It is noteworthy that no differences in VEGFR1 staining were noticed between *Ehd3^–/–^; Ehd4^–/–^* and control glomeruli. This is not surprising as VEGFR1 trafficking has not been shown to be regulated by endocytic regulators in the early or recycling pathway; hence we would not expect *Ehd3* and *Ehd4* deletion to affect trafficking of VEGFR1.

While we have not yet found a relevant endothelial cell system to analyze whether VEGFR2 distribution and expression pattern in *Ehd3^–/–^; Ehd4^–/–^* mice might reflect its altered endocytic traffic, studies of individual siRNA-mediated knock-down of EHD3 [26] or EHD4 [43] in HeLa cells indicate that they control traffic of transferrin receptor out of sorting endosomes to a recycling compartment and double knock-down of EHD3 and EHD4 has an identical effect (data not shown). As VEGFR2 has been shown to reside in a sorting endosomal compartment and to recycle to the cell surface upon VEGF stimulation [41], [42], absence of EHD3 and EHD4 in glomerular endothelial cells might be expected to impose a block to its recycling and thus disrupt its signaling.

Given the roles of EHD proteins in regulating endocytic recycling, it is likely that loss of EHD3 and EHD4 causes aberrant trafficking of key receptors such as VEGFR2, that are critical for maintenance of glomerular endothelial cell function and endothelial-cell dependent inter-cellular signaling critical for glomerular health. Thus, further studies using the experimental model described here should help link the basic cell biological processes of endocytic traffic, sorting and recycling of such receptors to the pathogenesis of endothelial injury which is an integral part of a number diseases of substantial human health importance.

## Materials and Methods

### Breeding and maintenance of mice colonies

Male and female *Ehd3* ^+/–^ mice were crossed to generate *Ehd3* ^+/+^, *Ehd3* ^+/–^ and *Ehd3* ^–/–^ mice. To generate *Ehd3* ^–/–^; *Ehd4* ^–/–^ mice, the previously described *Ehd4* ^–/–^ mice [28] were mated to *Ehd3* ^–/–^ mice to generate *Ehd3* ^+/–^; *Ehd4* ^+/–^ mice and these were intercrossed to give rise to *Ehd3* ^–/–^; *Ehd4* ^–/–^ mice. Animals were genotyped by tail PCR and ink-tattooed on toes for identification. Primer sequences for PCR genotyping are available on request. All experiments involving animals were approved by the University of Nebraska Medical Center Institutional Animal Care and Use Committee and carried out under the approved IACUC protocol number 07-061-FC12. All animals were treated humanely in accordance with institutional guidelines and that of the National Institutes of Health (NIH) Guide for the Care and Use of Laboratory Animals.

### Tissue staining

Formalin fixed, paraffin embedded, 3 µm kidney sections from mice of the indicated genotype and ages were processed as described previously [28] and H&E, PAS and JMS staining was performed using standard procedures by the Tissue sciences and Pathology and Microbiology core facilities. Immunostaining was performed as described previously [28]. The following antibodies were purchased commercially and used: CD31 (1∶50), WT-1 (1∶50), nephrin (1∶50), synaptopodin (1∶50), desmin (1∶50) and VEGFR2 (1∶50). Fluorescein labeled tomato lectin (from *L.esculentum*) was commercially purchased (Vector labs, FL-1171). For EHD protein staining, formalin fixed, 3 µm kidney sections were deparaffinized and immunostained as described previously. Polyclonal rabbit anti-EHD3 primary antibody was used at a 1∶200 dilution, while polyclonal rabbit anti-EHD1, EHD2 and EHD4 primary antibodies were used at a 1∶50 dilution in PBS/5% fetal bovine serum and a goat anti-rabbit Alexa Fluor 488 (or 594, Invitrogen) secondary antibody was used at 1∶200 dilution in PBS. The slides were mounted in Vectashield (Vector Labs) containing DAPI. Confocal images were acquired with a LSM510 fluorescence confocal microscope (Carl Zeiss, Thornwood, NY).

### Analyses of proteinuria

Urine from mice of indicated genotypes was assayed for proteinuria using a dip-stick method with Albustix (Siemens). Urine samples were boiled for 5 minutes in equal volumes of 2X sample buffer and 0.8 to 2 µl was loaded on a 7.5% SDS-PAGE gel. Bovine serum albumin (BSA) was used as a positive control. Following separation of proteins, the gel was stained using Coomassie Brilliant Blue and images were scanned following de-staining in water.

### Electron Microscopy

Kidneys were fixed in a 2.0% paraformaldehyde/2.5% glutaraldehyde phosphate buffered fixative, secondarily fixed with 1% osmium tetroxide, dehydrated using a graded acetone series and infiltrated using Polybed 812 epoxy resin. Blocks were polymerized at 60°C. Sections were thinned at 70 nm and stained using uranyl acetate and lead stains followed by scanning under a JEOL 1230 transmission electron microscope. Digital images were taken using a KeenView high-resolution camera and Soft Imaging Solutions AnalySIS ITEM digital software.

### TUNEL

For TUNEL assay, 3 µm neutral buffered formalin fixed kidney sections from d17, d23 and d45 *Ehd3^–/–^*; *Ehd4^–/–^* mice and littermate controls were deparaffinized and following antigen retrieval, an In Situ Cell Death Detection kit, POD (Roche) was used for TUNEL assay which was performed following the manufacturer's instructions. Appropriate negative and positive controls were included in each experiment. Confocal images of TUNEL were acquired with a LSM510 fluorescence confocal microscope (Carl Zeiss, Thornwood, NY) under either a 63X objective. TUNEL positive cells were counted from 12 sections (1 section/kidney, 2 kidneys/mice, 3 mice/genotype) and plotted.

## Supporting Information

Figure S1 **Generation and characterization of** ***Ehd3^–/–^*** **mice.** (A) Partial restriction map of the murine *Ehd3* locus, the targeting vector and the mutated *Ehd3* loci is depicted. *LoxP* sequences were inserted to flank the first exon such that it could be deleted by Cre/*loxP*-mediated recombination. Black rectangles represent exons; black and grey triangles represent *loxP* and *FRT* sequences, respectively. RI, *EcoR*I; H, HindIII. (B) Samples of tail DNA from 10 day old mice were genotyped by PCR. Three primers were used in a single duplex PCR reaction to amplify the WT *Ehd3* allele (377 bp) and the deleted allele (488 bp), and the products were separated by agarose gel electrophoresis to determine one of three genotypes of mice carrying various *Ehd3* alleles. (C) Western blotting of organ lysates from *Ehd3* mice. Fifty µg aliquots of organ lysates from three month-old *Ehd3* wild-type (*Ehd3* ^+/+^), heterozygote (E*hd3* ^+/–^) and null (*Ehd3* ^–/–^) male mice were subjected to Western blotting with antisera raised against human EHD proteins as described under Materials and Methods. The membrane was serially probed beginning with EHD3, followed by EHD1 and EHD4 and then EHD2 antibodies. The * denotes bleed-through from the previous blot. In the kidney and brain lysates, the anti-EHD3 antibody recognizes smaller sized products that may represent tissue-specific alternate spliced products of EHD3 lacking the first exon or they may be non-specific bands detected by the antibody. MWM, Molecular weight marker. (D and E) Quantitative growth curves of female (D) and male (E) littermate *Ehd3* mice (numbers in parentheses indicate number of mice of each genotype weighed).
(TIF)

Figure S2 **Lack of glomerular phenotypes in** ***Ehd3^–/–^*** **mice.** (A–D) Formalin-fixed, paraffin- embedded, three µm thick kidney sections from 3–5 month old male *Ehd3^+/+^* and *Ehd3^–/–^* mice were stained with H&E and PAS. (F–M) Immunostaining was performed on kidney sections using antibodies to synaptopodin (F–G), nephrin (H–I), WT-1(J–K) and desmin (L–M) as described in Materials and Methods. Scale bar = 10 µm. (E) Two µl of boiled urine samples from 8 month old mice of indicated genotypes were run on a 7.5% SDS-PAGE and stained using Coomassie Blue. Gels were scanned following de-staining. Bovine serum albumin (BSA) was used as a positive control (lanes 2 and 3). MWM  =  molecular weight marker (lane 1). Lanes 1–6 were run on the same gel but were noncontiguous. (N–O) Electron micrographs of glomeruli from 8 month old *Ehd3^+/+^* and *Ehd3^–/–^* mice are shown. Endothelium with fenestrations (black arrows) and podocytes with intact foot processes are seen in *Ehd3^+/+^* and *Ehd3^–/–^* mice. Scale bar = 2 µm.
(TIF)

Text S1 **Supporting Information Materials and Methods**
(DOC)
