# Supplementary figures and images for: Renal Thrombotic Microangiopathy in Mice with Combined Deletion of Endocytic Recycling Regulators EHD3 and EHD4
Source: PLoS One. 2011 Mar 9;6(3):e17838. doi: 10.1371/journal.pone.0017838 (PMC3052385; doi:10.1371/journal.pone.0017838)

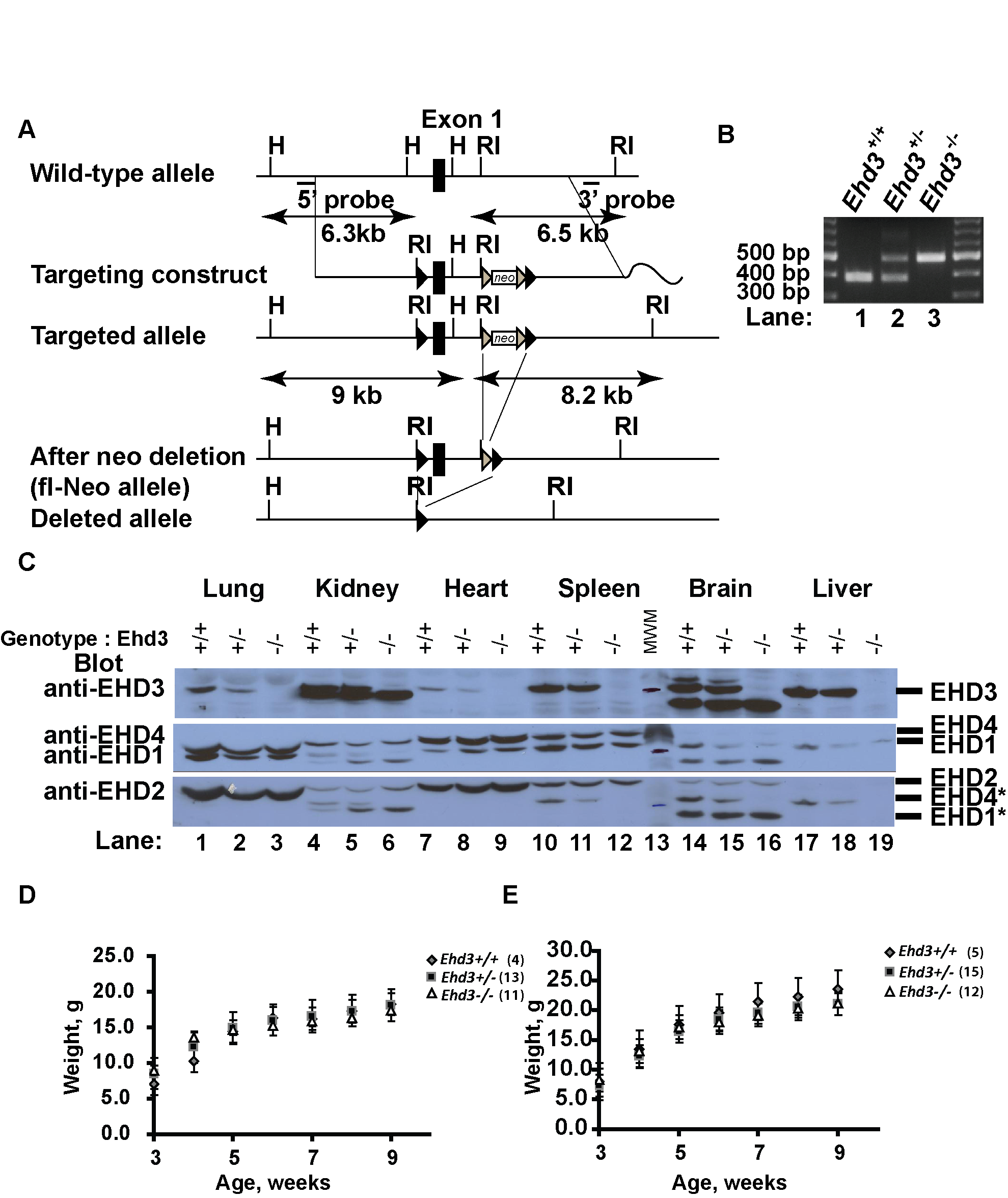

Supplement: Figure S1 — Generation and characterization of Ehd3–/– mice. (A) Partial restriction map of the murine Ehd3 locus, the targeting vector and the mutated Ehd3 loci is depicted. LoxP sequences were inserted to flank the first exon such that it could be deleted by Cre/loxP-mediated recombination. Black rectangles represent exons; black and grey triangles represent loxP and FRT sequences, respectively. RI, EcoRI; H, HindIII. (B) Samples of tail DNA from 10 day old mice were genotyped by PCR. Three primers were used in a single duplex PCR reaction to amplify the WT Ehd3 allele (377 bp) and the deleted allele (488 bp), and the products were separated by agarose gel electrophoresis to determine one of three genotypes of mice carrying various Ehd3 alleles. (C) Western blotting of organ lysates from Ehd3 mice. Fifty µg aliquots of organ lysates from three month-old Ehd3 wild-type (Ehd3 +/+), heterozygote (Ehd3 +/–) and null (Ehd3 –/–) male mice were subjected to Western blotting with antisera raised against human EHD proteins as described under Materials and Methods. The membrane was serially probed beginning with EHD3, followed by EHD1 and EHD4 and then EHD2 antibodies. The * denotes bleed-through from the previous blot. In the kidney and brain lysates, the anti-EHD3 antibody recognizes smaller sized products that may represent tissue-specific alternate spliced products of EHD3 lacking the first exon or they may be non-specific bands detected by the antibody. MWM, Molecular weight marker. (D and E) Quantitative growth curves of female (D) and male (E) littermate Ehd3 mice (numbers in parentheses indicate number of mice of each genotype weighed). (TIF) [file pone.0017838.s001.tif]

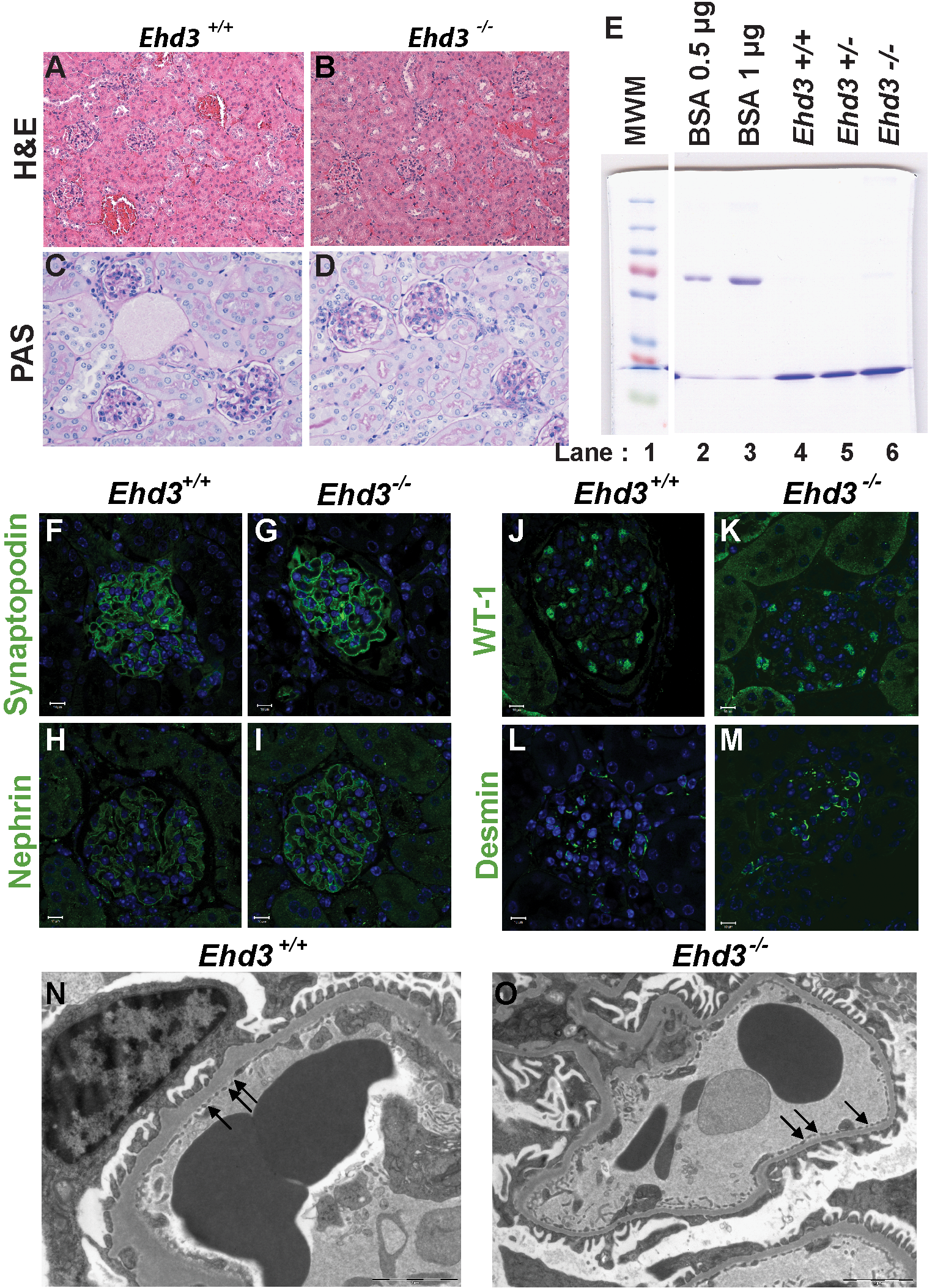

Supplement: Figure S2 — Lack of glomerular phenotypes in Ehd3–/– mice. (A–D) Formalin-fixed, paraffin- embedded, three µm thick kidney sections from 3–5 month old male Ehd3+/+ and Ehd3–/– mice were stained with H&E and PAS. (F–M) Immunostaining was performed on kidney sections using antibodies to synaptopodin (F–G), nephrin (H–I), WT-1(J–K) and desmin (L–M) as described in Materials and Methods. Scale bar = 10 µm. (E) Two µl of boiled urine samples from 8 month old mice of indicated genotypes were run on a 7.5% SDS-PAGE and stained using Coomassie Blue. Gels were scanned following de-staining. Bovine serum albumin (BSA) was used as a positive control (lanes 2 and 3). MWM = molecular weight marker (lane 1). Lanes 1–6 were run on the same gel but were noncontiguous. (N–O) Electron micrographs of glomeruli from 8 month old Ehd3+/+ and Ehd3–/– mice are shown. Endothelium with fenestrations (black arrows) and podocytes with intact foot processes are seen in Ehd3+/+ and Ehd3–/– mice. Scale bar = 2 µm. (TIF) [file pone.0017838.s002.tif]
