# Supplementary material for: Renal Thrombotic Microangiopathy in Mice with Combined Deletion of Endocytic Recycling Regulators EHD3 and EHD4
Source: PLoS One. 2011 Mar 9;6(3):e17838. doi: 10.1371/journal.pone.0017838 (PMC3052385; doi:10.1371/journal.pone.0017838)
Supplement: Text S1 — Supporting Information Materials and Methods (DOC) [file pone.0017838.s003.doc]

Supporting Information Materials and Methods

*Generation of Ehd3 gene-targeted mice*

A conditional gene knockout targeting vector was generated using the “recombineering” method (plasmids and bacterial strains provided by Dr. Neal G. Copeland, NCI, Frederick, Maryland). In brief, the NCBI Mouse Clone Finder was used to identify likely mouse *Ehd3* gene BAC clones and these were obtained from the Children’s Hospital Oakland Research Institute repository. PCR analysis showed the clone RP22-97B17 (derived from female 129S6/SvEvTAC mouse tissues) containing the *Ehd3* gene to be most suitable for further manipulations.

Using a series of “recombineering” reactions, an approximately 12 kb DNA fragment containing the first coding exon of *Ehd3* was retrieved into a plasmid and two *loxP* sites flanking this exon were introduced. A *FRT*-*Neo*-*FRT* selection cassette immediately preceding the second *loxP* site confers G418 resistance in transfected ES cells, with *FRT* sequences allowing removal of the cassette using FLP DNA recombinase; a single *FRT* and *loxP* sequence remained, keeping gene locus alterations to a minimum. PCR primer sequences used to generate the targeting vector and probes for Southern hybridization are available upon request. A *Not*I-linearized targeting vector was electroporated into the 129/Ola derived ES cell line HM1 and Southern hybridization using 5’ and 3’ external probes identified 2 out of 96 G418- and gancyclovir-selected clones to be correctly targeted; these were used to produce chimeric mice by blastocyst injection. One chimera achieved germline transmission of the targeted *Ehd3* allele.

To generate *Ehd3***^–/–^** mice, heterozygous *Ehd3*-targeted mice were mated with B6.FVB-Tg (EIIa-Cre) C5379Lmgd/J mice which express Cre recombinase from the adenovirus EIIa promoter for recombination in a wide range of tissues, including germ cells for transmission of the gene alteration to progeny. Heterozygous *Ehd3*-targeted, *cre* transgene-positive mice were crossed to C57BL/6J (wild-type) mice to generate heterozygous *Ehd3*-deleted, *cre* transgene-negative (*Ehd3*^+/-^) mice, which were used to produce *Ehd3*^–/–^ mice. All mice used in this study have been maintained on a mixed 129; C57BL/6J background.

*Harvesting of tissues and Western blotting*

Mice were euthanized using CO_2_ and organs were removed and tissue lysates prepared as described previously [28] for Western blotting. 100 μg aliquots of tissue lysates were separated using 8.5% SDS-PAGE and Western blotted using anti-EHD antibodies described previously. The antibody used to detect EHD3 is raised against a CNLKRMQDQLQAQ peptide and has been previously shown to be specific to EHD3 [28].
